# Supplementary material for: Human Sox4 facilitates the development of CXCL13-producing helper T cells in inflammatory environments
Source: Nat Commun. 2018 Sep 19;9:3762. doi: 10.1038/s41467-018-06187-0 (PMC6145936; doi:10.1038/s41467-018-06187-0)
Supplement: Supplementary file 1 — Supplementary Information [file 41467_2018_6187_MOESM1_ESM.pdf]

Supplementary Information for

Human Sox4 facilitates the development of CXCL13-producing helper T cells in inflammatory environments

Yoshitomi et al.

Supplementary Figures

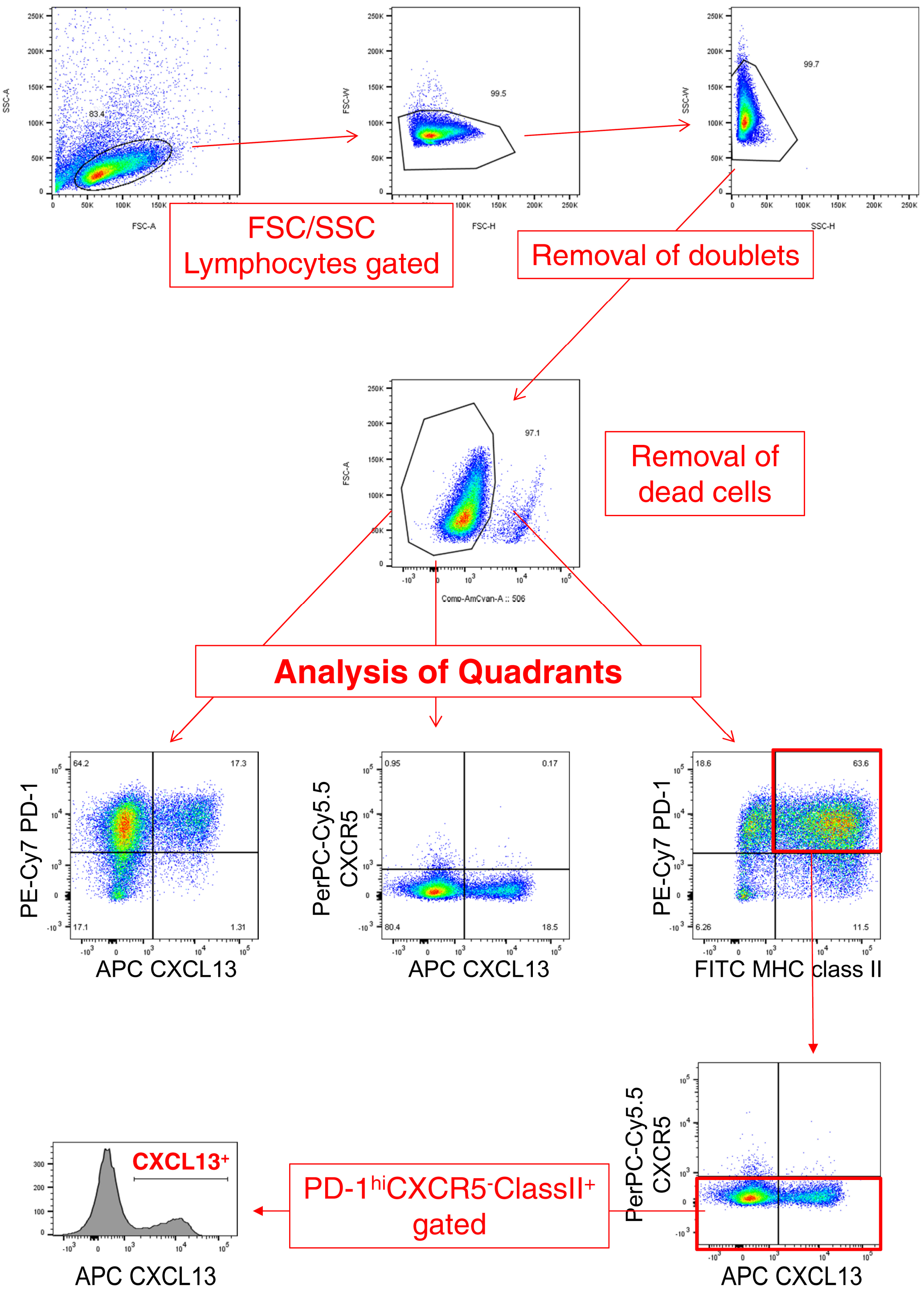

Supplementary Figure 1. Gating strategy of figure 1. Naïve human $CD4^+$ T cells differentiated for 5 days were gated with FSC and SSC, followed by removal of doublet cells and dead cells. Percentages of quadrants were investigated. As for $PD\text{-}1^{hi}CXCR5^{-}ClassII^{+}$ population, the frequency of CXCL13-positive cells in the population was investigated.

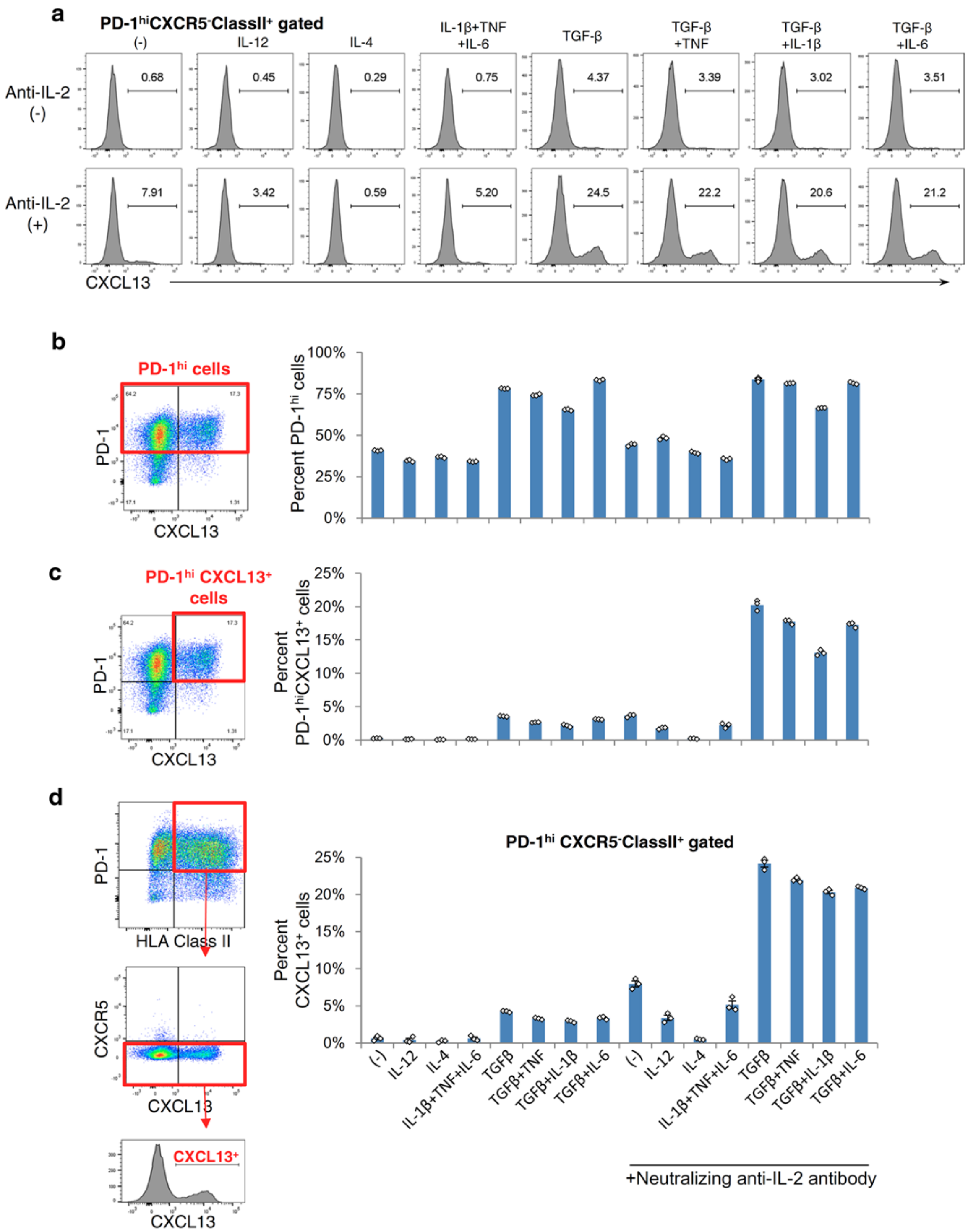

Supplementary Figure 2. Limiting of IL-2 upregulates CXCL13 production by PD-1^{hi}CXCR5^{-}ClassII^{+} cells. **a-d** Human naïve CD4^{+} T cells were differentiated as in figure 1. **a** Representative histograms showing frequency of CXCL13-positive cells in PD-1^{hi}CXCR5^{-}ClassII^{+} cells. **b-d** Percent PD-1^{hi} cells (**b**), percent PD-1^{hi}CXCL13^{+} cells (**c**), and percent CXCL13^{+} cells in PD-1^{hi}CXCR5^{-}ClassII^{+} cells (**d**) are shown as corresponding dots (n=3) and mean ± SEM with schematic definition of the populations.

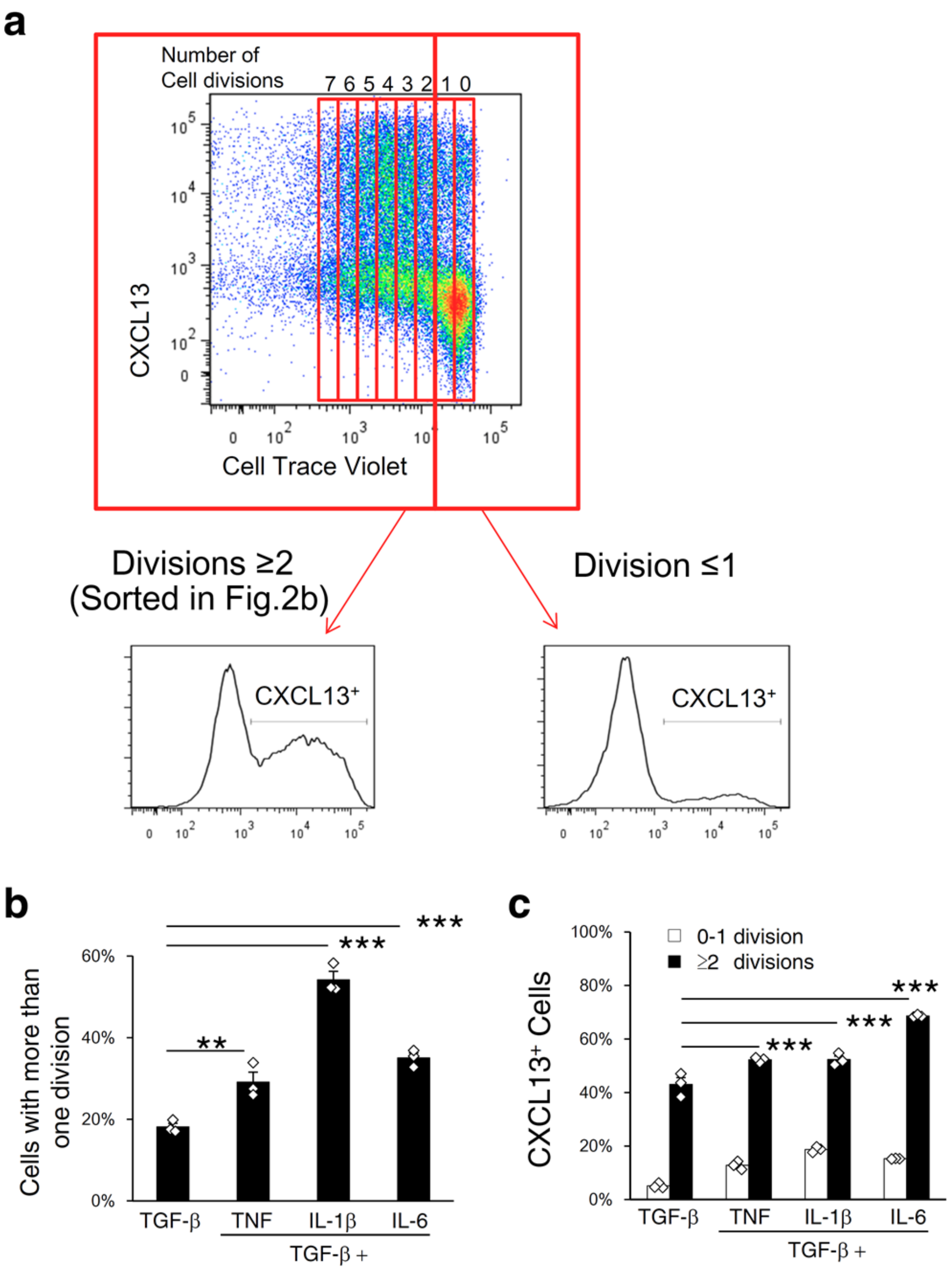

Supplementary Figure 3. Proinflammatory cytokines enhance cell proliferation and CXCL13 production. **a-c** Human blood CD4^{+} T cells labeled with CellTrace™ Violet were stimulated with plate-bound 10 µg/ml anti-CD3 and 10 µg/ml anti-CD28 antibodies in the presence of 10 µg/ml neutralizing anti-IL-2 antibody with TGF-β1 alone, TGF-β1 plus TNF, TGF-β1 plus IL-1β, and TGF-β1 plus IL-6 (each 10 ng/ml) for 5 days in serum-containing IMDM. **a** Schematic description of analysis. **b,c** Percentages of cells with more than one division in total cells (**b**), or percentages of CXCL13^{+} cells in cells with 0-1 division or in cells with more than one division (**c**) are shown. Quantifications were shown as corresponding dots (n=3) and mean ± SEM; $^{**}P < 0.01$, and $^{***}P < 0.001$ one-way ANOVA, Tukey test.

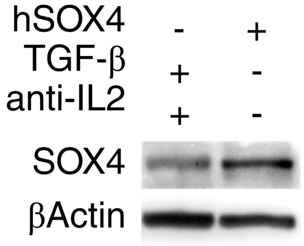

Supplementary Figure 4. Lentiviral induction of exogenous Sox4 protein in human CD4$^+$ T cells. Protein expression of Sox4 in human CD4$^+$ T cells cultured with TCR stimulation in the presence of TGF-β stimulation and IL-2-neutraliztion or lentiviral transduction of human Sox4. Sox4-transduced human CD4$^+$ T cells were sorted as YFP$^+$ cells.

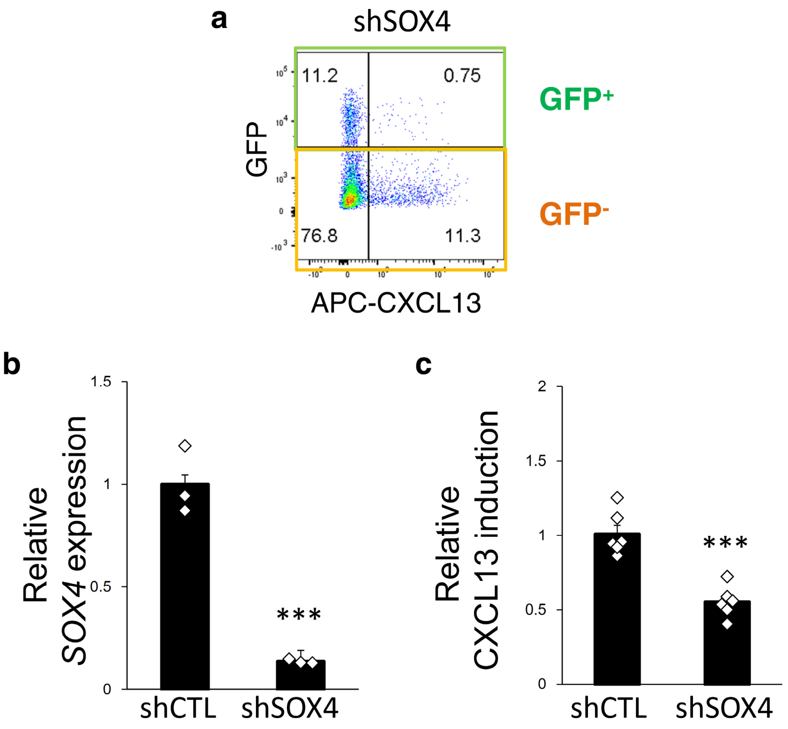

Supplementary Figure 5. Lentiviral knockdown of Sox4 significantly downregulated CXCL13 induction. **a-c** Naïve human $CD4^+$ T cells transduced with control or Sox4-specific shRNA by GFP-expressing lentivirus were differentiated in the presence of TGF-β. **a** Schematic description of analysis with a representative dot plot. **b,c** mRNA expression of *Sox4* relative to *18srRNA* determined by quantitative PCR in sorted GFP^+ cells (**b**, n=3), and relative induction of cells positive for CXCL13 protein in GFP^+ cells compared with GFP^- cells determined by flow cytometry (**c**, n=6 from two experiments) are shown. The data are presented as corresponding dots and mean ± SEM. ***P <0.001 unpaired student's t test.

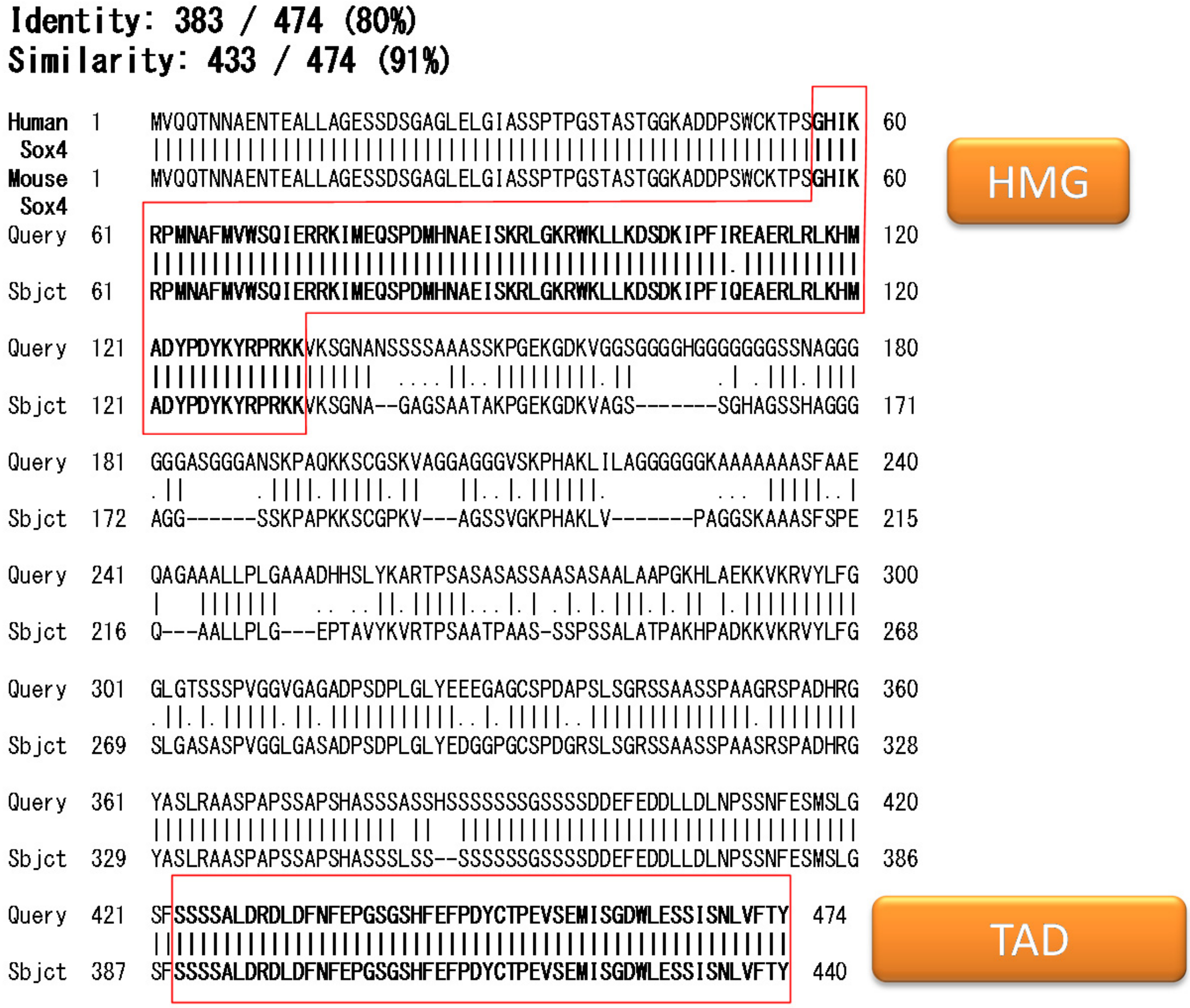

Supplementary Figure 6. Highly conserved similarity of Sox4 in humans and mice. Sox4 conserves high similarity especially in the N-terminus domain, HMG domain and TAD between humans and mice.

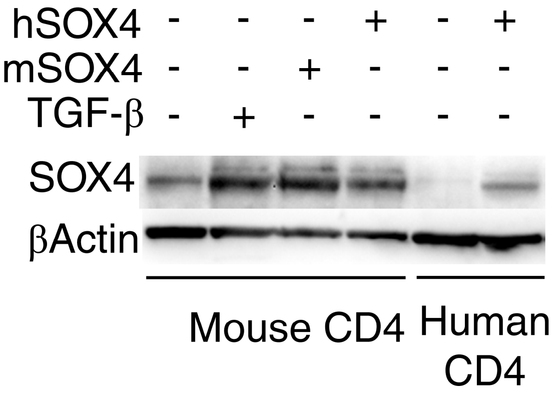

Supplementary Figure 7. Protein levels of Sox4 in mouse CD4$^+$ T cells. Mouse CD4$^+$ T cells were cultured with TCR stimulation in the presence or absence of TGF-β stimulation or lentiviral transduction of mouse Sox4 or human Sox4 as in figure 4d. As control, human naïve CD4$^+$ T cells without or with transduction of human Sox4 were also investigated. Sox4-transduced mouse and human CD4$^+$ T cells were sorted as YFP$^+$ cells. Expression of mouse or human Sox4 proteins were visualized by immunoblotting with antibodies harboring reactivity against both human and mouse. TGF-β stimulation and lentiviral transduction of mouse or human Sox4 in mouse CD4$^+$ T cells induced SOX4 expression comparable with the level in human Sox4-transduced human CD4$^+$ T cells which show CXCL13 induction as in figure 3.

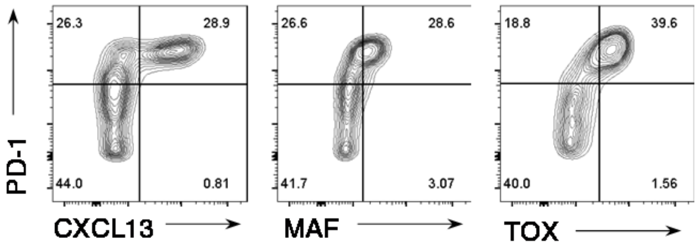

Supplementary Figure 8. RA synovial PD-1^{hi}CD4^{+} T cells preferentially express MAF and TOX. Representative density plots of RA synovial fluid CD4^{+} T cells stained with anti-PD-1, CXCL13, MAF or TOX antibodies. PD-1^{hi} cells preferentially express CXCL13, MAF and TOX.

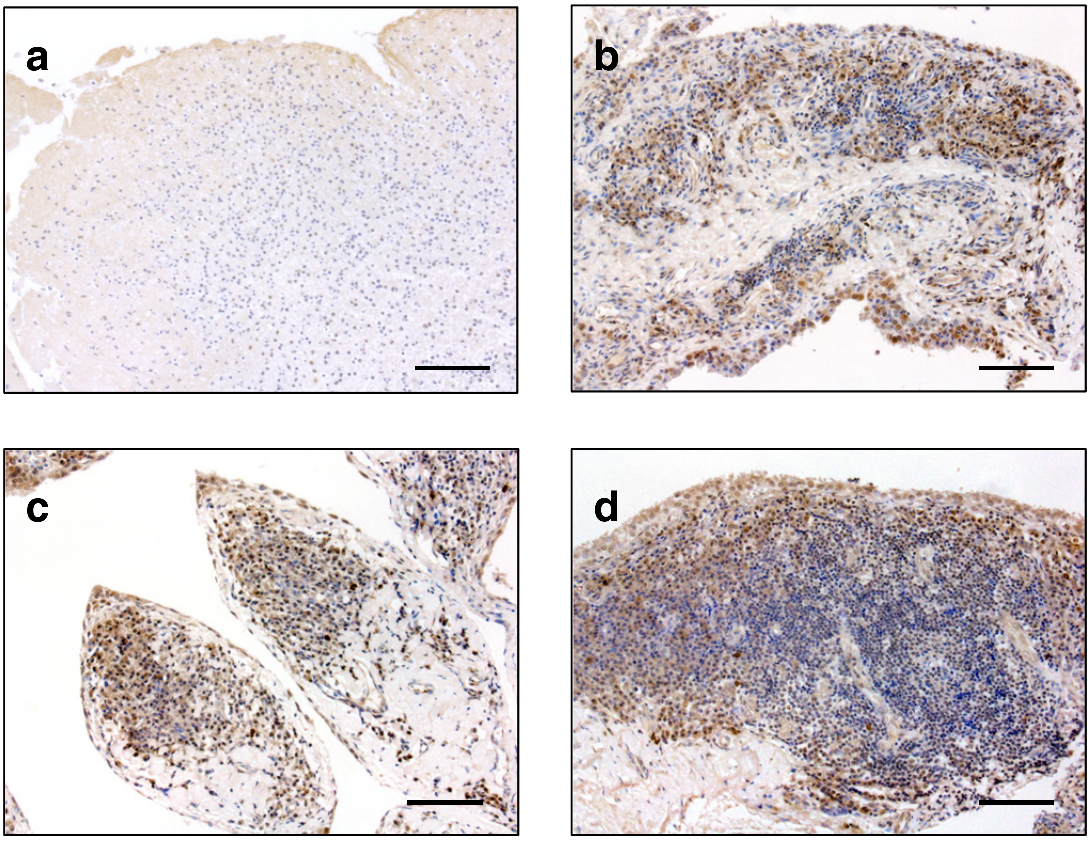

Supplementary Figure 9. Immunohistochemical staining with Sox4 antibody of RA synovial tissues. **a–d** Representative staining patterns of Sox4 in RA synovium. Sox4 (brown) and hematoxylin (blue). **a** A representative sample with moderate diffuse cell infiltration without ELS formation. $Sox4^{+}$ cells are not detectable. **b** A representative sample with moderate cell infiltration that forms mild nodule-like structures in sublining layer. Sox4 was preferentially expressed in nodule-like structures. **c** A representative sample with severe cell infiltration with ELS formation in villous hypertrophy of synovium. **d** A representative sample with severe cell infiltration with ELSs. Scale bars: 100 μm.

a

b

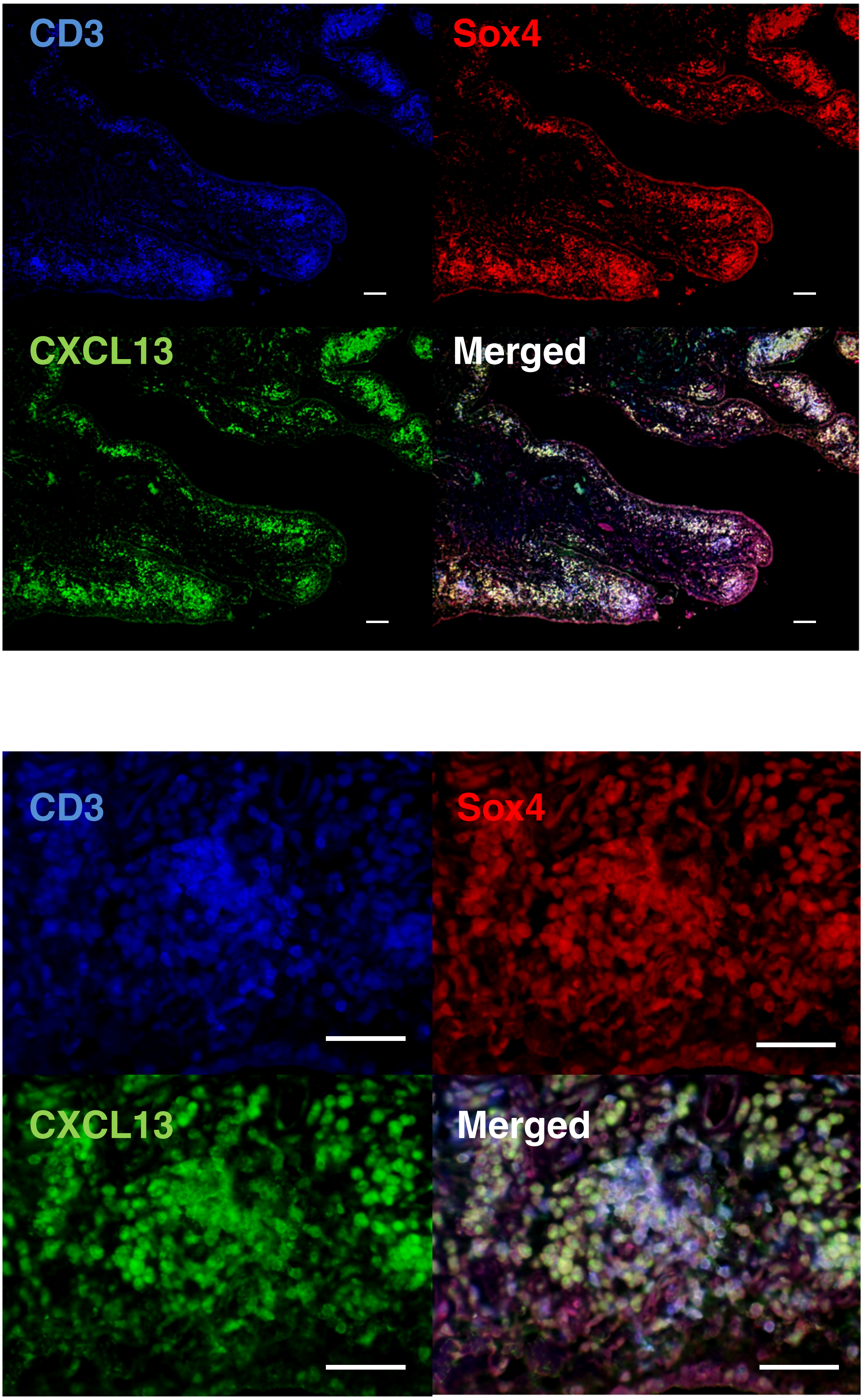

c

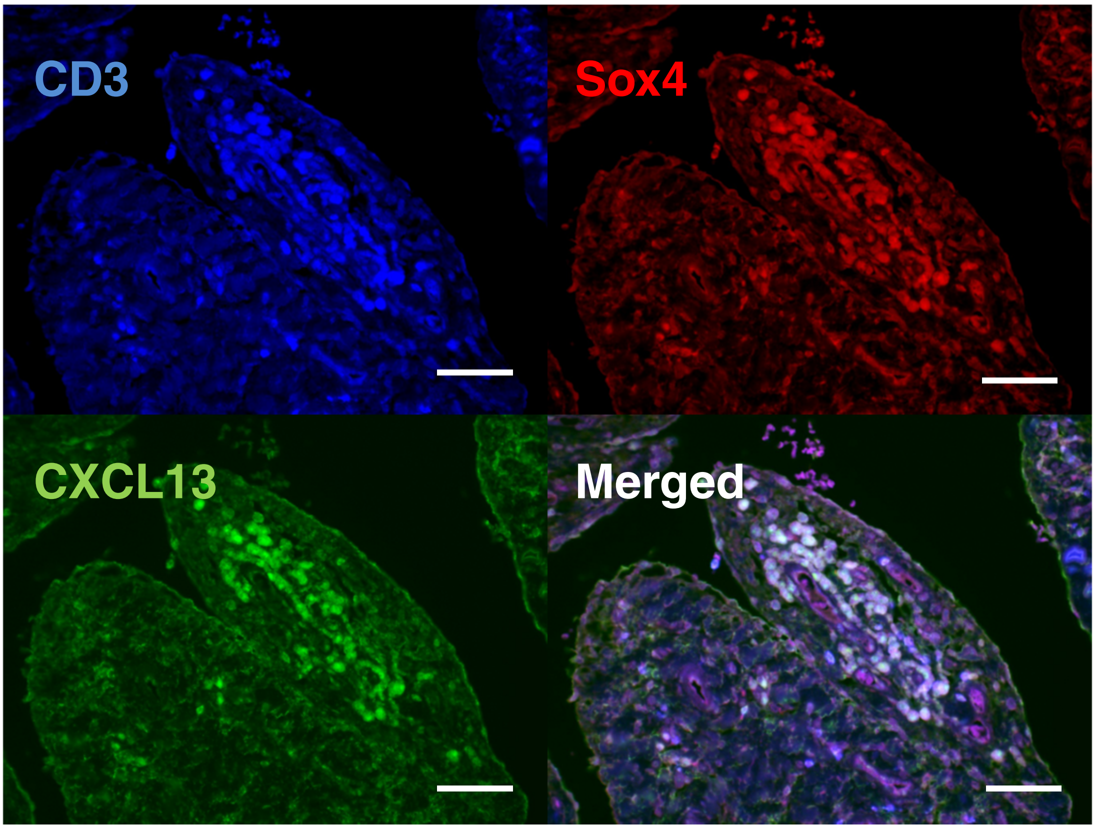

d

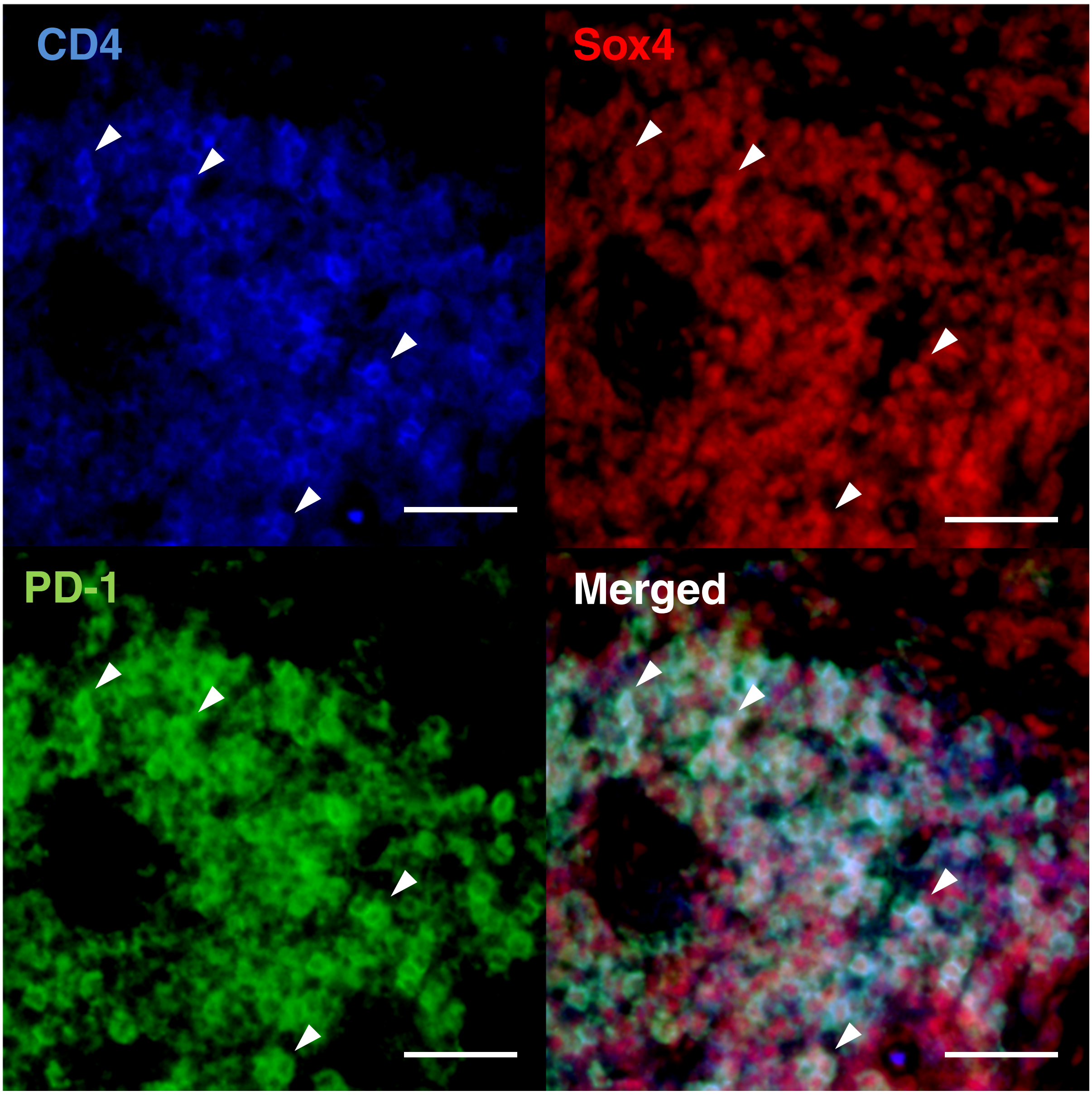

e

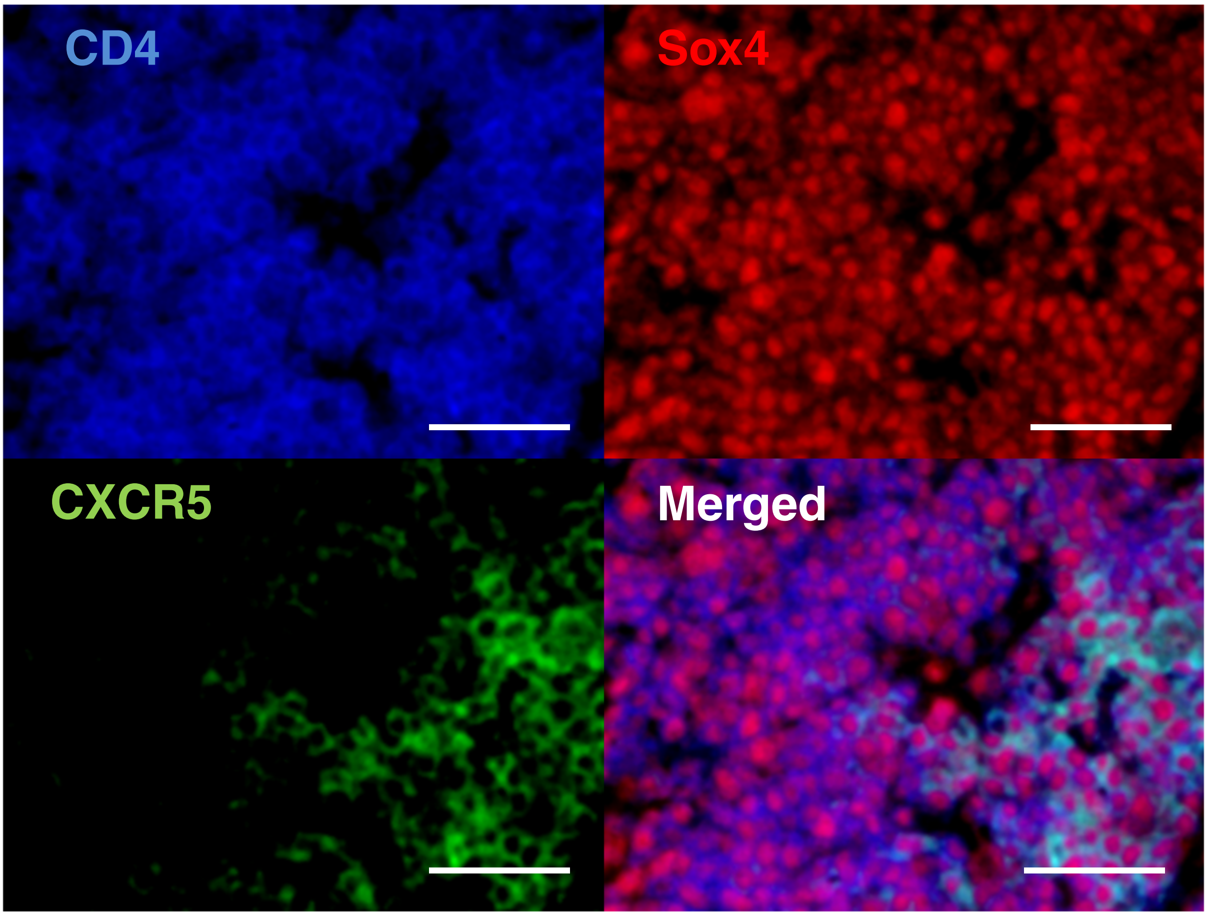

Supplementary Figure 10. Triple immunostaining of RA synovial tissues. **a–c** Stainings of CXCL13 (Green), Sox4 (Red), and CD3 (Blue). **a** A representative sample with moderate cell infiltration in sublining layer accompanied with moderate ELS formation. Sox4^{+} cells are present in both areas with diffuse cell infiltration and with ELS formation. Scale bars: 100 µm. **b** A representative sample with mild ELS formation. Scale bars: 50µm. **c** A representative sample of villous hyperplasia. Scale bars: 50 µm. **d** Staining of PD-1 (Green), Sox4 (red) and CD4 (Blue). Arrowheads indicate representative PD-1^{hi}CD4^{+}Sox4^{+} cells. Scale Bars: 50µm. **e** Staining of CXCR5 (Green), Sox4 (red) and CD4 (Blue). Scale Bars: 50µm.

Supplementary Figure 11. Full images of Immunoblots

Fig. 2d SOX4

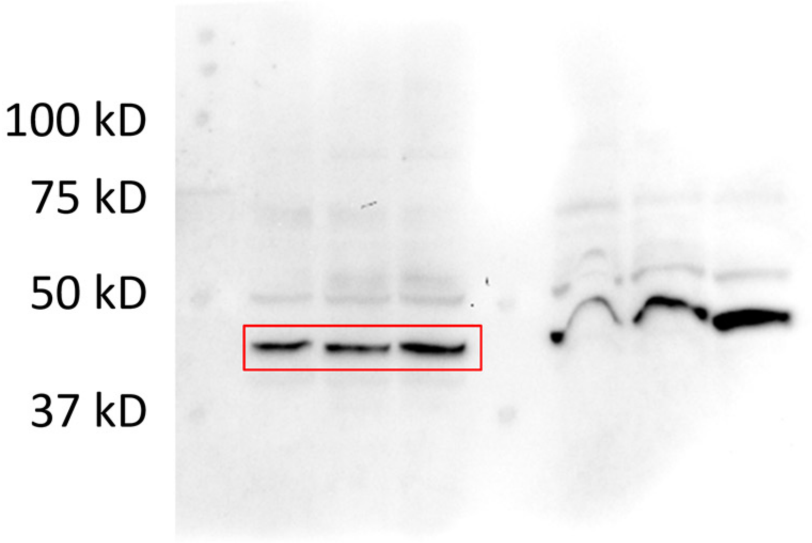

Fig. 2d ACTB

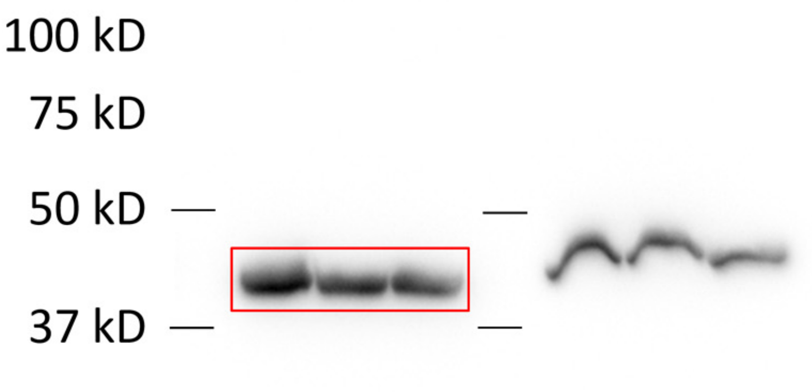

Supplementary Tables

Supplementary Table 1. Expressions of genes relating to Tfh cells, PD-$1^{hi}$$CXCR5^{-}$$CD4^{+}$ T cells of RA, and/or Th17 cells

Probe Set ID	Gene Symbol	Entrez Gene	FC (TGFβ + IL-1β vs IL-12)	FC (TGFβ + IL-6 vs IL-12)
205242_at	CXCL13	10563	32.0	38.2
203140_at	BCL6	604	-1.4	-1.5
215990_s_at	BCL6	604	-1.4	-1.5
228758_at	BCL6	604	-2.7	-2.8
207607_at	ASCL2	430	1.0	-1.3
229215_at	ASCL2	430	1.0	1.0
205965_at	BATF	10538	-1.5	1.4
206363_at	MAF	4094	1.9	5.4
209348_s_at	MAF	4094	2.7	3.3
1566324_a_at	MAF	4094	3.0	3.1
209347_s_at	MAF	4094	3.7	2.0
1566323_at	MAF	4094	1.1	1.2
204529_s_at	TOX	9760	2.6	6.3
204530_s_at	TOX	9760	1.2	2.1
217192_s_at	PRDM1	639	1.3	-1.2
235668_at	PRDM1	639	1.2	-1.1
228964_at	PRDM1	639	4.0	-1.1
206126_at	CXCR5	643	-2.3	-1.2
216734_s_at	CXCR5	643	-1.4	-1.2
210439_at	ICOS	29851	-1.4	-1.5
207634_at	PDCD1	5133	1.0	1.0
207892_at	CD40LG	959	-1.8	-1.8
209582_s_at	CD200	4345	1.6	1.1
209583_s_at	CD200	4345	1.6	1.0
240070_at	TIGIT	201633	2.6	3.4
211211_x_at	SH2D1A	4068	2.6	3.1
211209_x_at	SH2D1A	4068	2.8	3.0
211210_x_at	SH2D1A	4068	2.7	3.0
210116_at	SH2D1A	4068	2.4	2.9
230391_at	CD84	8832	3.0	2.2
211188_at	CD84	8832	-1.4	-2.1
205988_at	CD84	8832	1.7	1.5
244352_at	CD84	8832	1.6	1.4
211192_s_at	CD84	8832	1.7	1.4
211190_x_at	CD84	8832	1.7	1.3
211189_x_at	CD84	8832	1.7	1.2
211191_at	CD84	8832	1.1	-1.2
1552497_a_at	SLAMF6	114836	1.3	-1.0
206181_at	SLAMF1	6504	-1.8	-1.6
1555626_a_at	SLAMF1	6504	-1.5	-1.4
239427_at	SLAMF1	6504	-1.1	1.0
221271_at	IL21	59067	-11.4	-20.2
206978_at	CCR2	729230	5.5	4.1
207794_at	CCR2	729230	1.9	1.5
200839_s_at	CTSB	1476	-1.5	-2.0
200838_at	CTSB	1476	-1.5	-1.9
213275_x_at	CTSB	1476	-1.2	1.6
227961_at	CTSB	1476	-1.6	1.5
213274_s_at	CTSB	1476	-1.5	-1.1

Supplementary Table 1. Continued

Probe Set ID	Gene Symbol	Entrez Gene	FC (TGFβ + IL-1β vs IL-12)	FC (TGFβ + IL-6 vs IL-12)
206419_at	RORC	6097	1.2	1.2
228806_at	RORC	6097	2.1	1.9
210426_x_at	RORA	6095	1.1	1.2
210479_s_at	RORA	6095	1.1	1.2
226682_at	RORA	6095	1.7	1.7
235567_at	RORA	6095	1.5	1.6
236266_at	RORA	6095	2.7	3.5
240951_at	RORA	6095	1.0	1.4
216876_s_at	IL17A	3605	2.0	4.3
234408_at	IL17F	112744	-1.0	-1.0
221165_s_at	IL22	50616	-4.6	-4.5
222974_at	IL22	50616	-51.2	-30.7
1552912_a_at	IL23R	149233	-1.4	-3.7
1561853_a_at	IL23R	149233	-1.2	-2.9

Supplementary Table 2. Candidate genes that fulfilled the criteria

Probe Set ID	Gene Symbol	Entrez Gene	FC (TGF-β +IL-1β vs IL-12)	FC (TGF-β +IL-6 vs IL-12)	Localization
219529_at	CLIC3	9022	14.7	12.9	Nucleus
218318_s_at	NLK	51701	4.3	6.4	Nucleus
201416_at	SOX4	6659	4.6	4.9	Nucleus
201417_at	SOX4	6659	6.9	6.8	Nucleus
202087_s_at	CTSL	1514	24.2	43.0	Cytoplasm
228739_at	CYS1	192668	21.8	20.6	Cytoplasm
206588_at	DAZL	1618	18.6	45.3	Cytoplasm
215017_s_at	FNBP1L	54874	17.1	26.6	Cytoplasm
225700_at	GLCCI1	113263	4.5	4.8	Cytoplasm
1560316_s_at	GLCCI1	113263	4.2	4.9	Cytoplasm
205466_s_at	HS3ST1	9957	7.7	19.1	Cytoplasm
227889_at	LPCAT2	54947	10.0	27.9	Cytoplasm
227556_at	NME7	29922	16.6	20.7	Cytoplasm
213010_at	PRKCDBP	112464	7.8	11.9	Cytoplasm
217762_s_at	RAB31	11031	12.2	5.4	Cytoplasm
217763_s_at	RAB31	11031	10.0	4.2	Cytoplasm
217764_s_at	RAB31	11031	15.4	7.8	Cytoplasm
209324_s_at	RGS16	6004	4.9	4.0	Cytoplasm
209325_s_at	RGS16	6004	6.2	5.1	Cytoplasm
204067_at	SUOX	6821	10.6	13.0	Cytoplasm
1553030_a_at	SUOX	6821	4.5	4.4	Cytoplasm
204604_at	CDK14	5218	4.6	6.8	Cytoplasm
201525_at	APOD	347	8.3	6.8	Extracellular Space
205229_s_at	COCH	1690	12.0	5.9	Extracellular Space
213428_s_at	COL6A1	1291	7.5	12.2	Extracellular Space
205242_at	CXCL13	10563	30.6	38.1	Extracellular Space
226847_at	FST	10468	11.8	9.4	Extracellular Space
243110_x_at	NPW	283869	8.1	8.2	Extracellular Space
211756_at	PTHLH	5744	4.6	5.9	Extracellular Space
206310_at	SPINK2	6691	10.7	12.8	Extracellular Space
213994_s_at	SPON1	10418	16.3	12.9	Extracellular Space
201506_at	TGFBI	7045	16.8	44.0	Extracellular Space
223502_s_at	TNFSF13B	10673	21.0	21.3	Extracellular Space
207426_s_at	TNFSF4	7292	14.6	33.9	Extracellular Space
227233_at	TSPAN2	10100	6.2	5.3	Extracellular Space
227236_at	TSPAN2	10100	5.8	5.7	Extracellular Space
1563445_x_at	CTSLP8	1518	8.8	9.1	Other
208029_s_at	LAPTM4B	55353	5.9	8.0	Other
208767_s_at	LAPTM4B	55353	5.9	7.5	Other
214039_s_at	LAPTM4B	55353	5.6	6.9	Other
1554679_a_at	LAPTM4B	55353	7.2	8.6	Other
230650_at	SLCO5A1	81796	14.0	28.1	Other
213851_at	TMEM110	375346	4.4	6.5	Other
235079_at	ZNF704	619279	4.1	4.4	Other
202391_at	BASP1	10409	14.5	35.0	Other
219010_at	C1orf106	55765	7.9	13.5	Other
227966_s_at	CCDC74A///CCDC74B	90557///91409	4.0	5.3	Other
214079_at	DHRS2	10202	10.8	24.8	Other

Supplementary Table 2. Continued

Probe Set ID	Gene Symbol	Entrez Gene	FC (TGF-β +IL-1β vs IL-12)	FC (TGF-β +IL-6 vs IL-12)	Localization
1554918_a_at	ABCC4	10257	9.0	7.7	Plasma Membrane
201242_s_at	ATP1B1	481	21.9	34.5	Plasma Membrane
201243_s_at	ATP1B1	481	14.7	24.7	Plasma Membrane
231873_at	BMPR2	659	5.8	5.5	Plasma Membrane
203065_s_at	CAV1	857	9.8	15.0	Plasma Membrane
212097_at	CAV1	857	20.6	44.8	Plasma Membrane
204440_at	CD83	9308	5.5	10.4	Plasma Membrane
201131_s_at	CDH1	999	4.8	9.0	Plasma Membrane
226189_at	ITGB8	3696	9.5	20.9	Plasma Membrane
229461_x_at	NEGR1	257194	21.9	33.3	Plasma Membrane
221796_at	NTRK2	4915	7.6	24.2	Plasma Membrane
210830_s_at	PON2	5445	4.5	8.2	Plasma Membrane
209611_s_at	SLC1A4	6509	6.5	6.2	Plasma Membrane
225688_s_at	PHLDB2	90102	5.0	5.5	Plasma Membrane
207536_s_at	TNFRSF9	3604	4.9	4.5	Plasma Membrane
211786_at	TNFRSF9	3604	6.3	5.6	Plasma Membrane
210643_at	TNFSF11	8600	9.2	10.5	Plasma Membrane
235438_at			4.3	6.2	

Supplementary Table 3. Clinical characteristics of RA participants

Donors of paired blood and synovial CD4$^+$ T cells	
Number of participants	14
Gender, male/female	4/10
Age (year)	67.8±9.6
Disease Duration (years)	14.4±10.6
Positive RF, n (%)	13 (92.9%)
Positive ACPA, n (%)	12 (85.7%)
ESR, mm/hour	38.8±24.2
C-reactive protein, mg/liter	18.2±23.0
DAS28, Average	4.77±1.15
Medication, n (%)	10 (100%)
Steroid	4 (28.6%)
Synythetic DMARDs	13 (92.9%)
Biologics	6 (42.9%)

Totally 10 samples of synovial fluid and 5 samples of synovial tissue were investigated.Both syonivial fluid and synovial tissue were available from one participant, whose data of pripheral blood CD4$^+$ T cells was used in both comparisons for synovial fluid and synovial tissue.

Paraffin-embedded RA synovial tissues	
Number of samples	14
Gender, male/female	2/12
Age (year)	58.8±17.0
Disease Duration (years)	13.0±13.3
Positive RF, n (%)	12 (85.7%)
Positive ACPA, n (%)	11 (78.6%)
ESR, mm/hour	46.9±30.3
C-reactive protein, mg/liter	20.7±20.6
DAS28, Average	4.01±1.39
Medication, n (%)	13 (92.9%)
Steroid	6 (42.9%)
Synythetic DMARDs	11 (78.6%)
Biologics	6 (42.9%)

SD, standard deviatin; RF, rheumatoid factor; ACPA, anti-citrullinated protein antibody; ESR, erythrocyte sedimaentation ratio; DAS28, 28-joint Disease Activity Score, DMARDs, Disease Modifying Anti-rheumatic Drugs.
Age, ESR, C-reactive protein and DAS28 are shown as mean± SD.
